# Transcriptional Profiling Identifies Upregulation of Neuroprotective Pathways in Retinitis Pigmentosa

**DOI:** 10.3390/ijms22126307

**Published:** 2021-06-11

**Authors:** Christina B. Bielmeier, Saskia Roth, Sabrina I. Schmitt, Stefaniya K. Boneva, Anja Schlecht, Mario Vallon, Ernst R. Tamm, Süleyman Ergün, Andreas Neueder, Barbara M. Braunger

**Affiliations:** 1 Institute of Anatomy and Cell Biology, Julius-Maximilians-University Wuerzburg, Koellikerstr. 6, D-97070 Würzburg, Germany; Christina.Bielmeier@uni-wuerzburg.de (C.B.B.); Saskia.Roth@uni-wuerzburg.de (S.R.); anja.schlecht@uni-wuerzburg.de (A.S.); mario.vallon@uni-wuerzburg.de (M.V.); sueleyman.erguen@uni-wuerzburg.de (S.E.); 2 Institute of Human Anatomy and Embryology, University of Regensburg, D-93053 Regensburg, Germany; sabrina.schmitt@ur.de (S.I.S.); ernst.tamm@ur.de (E.R.T.); 3 Eye Center, Medical Center, Faculty of Medicine, University of Freiburg, D-79078 Freiburg, Germany; stefaniya.boneva@uniklinik-freiburg.de; 4 Department of Neurology, University of Ulm, D-89069 Ulm, Germany; andreas.neueder@uni-ulm.de

**Keywords:** retinitis pigmentosa, VPP mouse model, in-situ hybridization, neurodegeneration, neuroinflammation, extracellular matrix disorganisation, neuroprotective pathways

## Abstract

Hereditary retinal degenerations like retinitis pigmentosa (RP) are among the leading causes of blindness in younger patients. To enable in vivo investigation of cellular and molecular mechanisms responsible for photoreceptor cell death and to allow testing of therapeutic strategies that could prevent retinal degeneration, animal models have been created. In this study, we deeply characterized the transcriptional profile of mice carrying the transgene rhodopsin V20G/P23H/P27L (VPP), which is a model for autosomal dominant RP. We examined the degree of photoreceptor degeneration and studied the impact of the VPP transgene-induced retinal degeneration on the transcriptome level of the retina using next generation RNA sequencing (RNASeq) analyses followed by weighted correlation network analysis (WGCNA). We furthermore identified cellular subpopulations responsible for some of the observed dysregulations using in situ hybridizations, immunofluorescence staining, and 3D reconstruction. Using RNASeq analysis, we identified 9256 dysregulated genes and six significantly associated gene modules in the subsequently performed WGCNA. Gene ontology enrichment showed, among others, dysregulation of genes involved in TGF-β regulated extracellular matrix organization, the (ocular) immune system/response, and cellular homeostasis. Moreover, heatmaps confirmed clustering of significantly dysregulated genes coding for components of the TGF-β, G-protein activated, and VEGF signaling pathway. 3D reconstructions of immunostained/in situ hybridized sections revealed retinal neurons and Müller cells as the major cellular population expressing representative components of these signaling pathways. The predominant effect of VPP-induced photoreceptor degeneration pointed towards induction of neuroinflammation and the upregulation of neuroprotective pathways like TGF-β, G-protein activated, and VEGF signaling. Thus, modulation of these processes and signaling pathways might represent new therapeutic options to delay the degeneration of photoreceptors in diseases like RP.

## 1. Introduction

Retinitis pigmentosa (RP) is a hereditary form of retinal degeneration that results from mutations in any one of more than 70 known susceptibility genes [1,2]. Quite intriguingly, RP is considered as one of the most common hereditary diseases associated with mutations in core splicing proteins resulting in the altered regulation of gene expression [2]. Even though RP is considered a rare genetic disorder, it is still among the major causes of blindness in younger patients [3,4], caused by the progressive loss of rod and cone photoreceptor cells, respectively [1]. Photoreceptors are the light sensitive neurons of the retina that are responsible for visual perception [5]. These cells consist of the outer and inner segments, which are connected through a cilium with the cell’s perikaryal, located in the outer nuclear layer (ONL). They form a synaptic layer in the outer plexiform layer (OPL) of the retina to signal to the inner retinal neurons [4,5]. Photoreceptor degeneration typically results in a thinning of the ONL concomitant with the loss of the inner and outer photoreceptor segments, resulting in an impaired visual function up to blindness. Based on the genetic heterogeneity of RP, it is still a challenge to understand and, more importantly, to inhibit the molecular mechanisms leading to the degeneration of photoreceptors, with the overall goal of delaying it. Consequently, animal models mimicking photoreceptor degeneration are frequently used to gain insights into the impact of certain mutations on the degeneration of photoreceptors. Among these, the VPP mouse model is a well-known animal model for photoreceptor degeneration. VPP mice carry a rhodopsin transgene with three amino acid substitutions: Val-20 → Gly (V20G), Pro-23 → His (P23H), and Pro-27 → Leu (P27L) (VPP) [6], with the P23H mutation being the most prevalent mutation in U.S. patients suffering from autosomal dominant RP [7,8,9]. Hemizygous VPP mice harbor two to five copies of the mutant rhodopsin transgene at a single integration site in addition to the wildtype rhodopsin gene [6]. Transgene expression results in slowly progressing degeneration of rod and cone photoreceptors [6]. In the current study, we first analyzed the morphology and apoptotic events in retinae of VPP mice. In humans, the various RP mutations of core splicing proteins result in an altered transcriptome [2]. Therefore, we furthermore asked the question whether mutations of other genes, e.g., the rhodopsin gene, might also influence the retinal transcriptome with the overall aim to identify molecular key factors and signaling pathways that predominantly influence the course of photoreceptor degeneration. Thus, we studied the impact of the VPP mutation on the retinal transcriptome using RNASeq analyses, which has not been done so far. In summary, we identified more than 9000 dysregulated genes. By performing gene correlation network analyses, we identified six significantly associated modules in VPP animals. Gene ontology enrichment analyses showed, among others, involvement of components of the (ocular) immune system or response, respectively; transforming growth factor ß (TGF-β) regulated extracellular matrix organization; and dysregulation of the cellular homeostasis. The progressive photoreceptor loss was highlighted by, e.g., reduced expression of photoreceptor specific transcripts, a downregulation of the rhodopsin mediated signaling pathway, and the reactivity of (micro)glial cells. In summary, our findings show that apoptosis; neuroinflammation; and the upregulation of neuroprotective pathways like TGF-β, endothelin-, and vascular endothelial growth factor (VEGF)-signaling are among the dominant effects following hereditary retinal degeneration in the VPP retina.

## 2. Results

### 2.1. Photoreceptor Degeneration in VPP Mice

To validate the VPP model, we quantified TUNEL-positive, apoptotic cells of one-month-old animals (Supplementary Figure S1A–C; controls: 16.28 ± 3.08 apoptotic cells/mm² ONL, VPP: 173.57 ± 17.84 apoptotic cells/mm² ONL, *p* < 0.001) and performed morphometric analyses on semithin sections of the eyes of three-month-old control and VPP animals (Supplementary Figure S1D–F) to show the beginning and more progressed photoreceptor degeneration in VPP mice [6]. We furthermore analyzed mRNA expression levels by qPCR of factors like *leukemia inhibitory factor* (*Lif*), *fibroblast growth factor 2* (*Fgf2*), and *endothelin 2* (*Edn2*) that are well-known to be upregulated in the context of retinal degeneration [10,11,12] and found them to be significantly upregulated in retinae of three-month-old VPP mice (*Lif*: 22.38 ± 4.13, *p* = 0.008; *Fgf2*: 13.17 ± 1.52, *p* = 0.005; *Edn2*: 46.33 ± 5.64, *p* < 0.001) compared with retinae of control littermates (*Lif*: 1.00 ± 0.18; *Fgf2*: 1.00 ± 0.14; *Edn2:* 1.00 ± 0.15) (Supplementary Figure S1G).

### 2.2. Transcriptional Alterations in VPP Retinae: RNAseq and Weighted Correlation Network Analysis (WGCNA)

**Figure 1:**
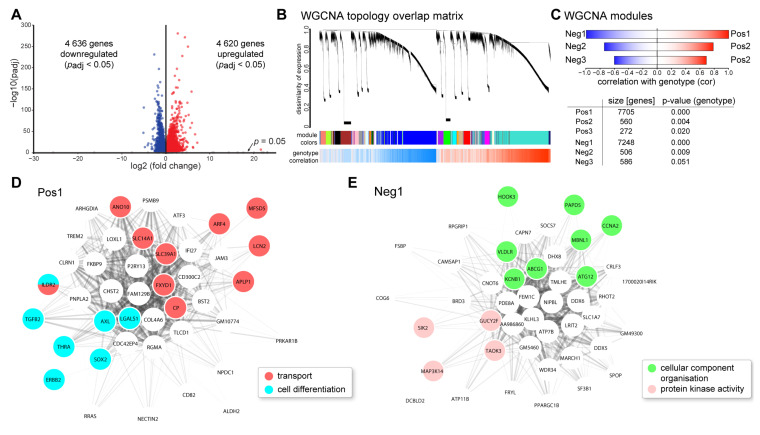
**Transcriptome analysis of VPP mice:** (**A**) RNAseq analysis of three-month-old identified more than 4600 significantly down- and up-regulated genes, respectively (Benjamini–Hochberg adjusted *p*-values; *p_adj_*). (**B**) Weighted correlation network analysis (WGCNA) showed large clusters of genes (modules) that were positively or negatively correlated with the genotype. Blue color in the panel below indicates lower expression and red color indicates higher expression in the VPP mice. (**C**) For each sign of correlation, three significantly correlated modules that changed in the VPP mice were identified. (**D**,**E**) Intra-module analysis of the Pos1 (**D**) and Neg1 (**E**) modules. The 50 highest connected (intramodular connectivity) genes are shown. Coloring of the genes corresponds to significantly enriched gene ontology terms.

Subsequently, we applied next generation RNA sequencing (RNAseq) analyses to investigate the impact of VPP transgene expression and concomitant photoreceptor degeneration on the transcriptome of the retina in three-month-old VPP and control animals. Out of the total of 54,532 genes in the Ensembl gene annotation for mouse (Mus musculus GRCm38 v. 94), we found 30,796 genes to be expressed in the retina, of which 9256 were dysregulated (4636 down- and 4620 upregulated, Figure 1A, cut off criteria: Benjamin-Hochberg adjusted *p*-value (*p_adj_*) < 0.05). The top 30 dysregulated genes are shown in Supplementary Table S2. Among others, genes regulating processes in neurotransmission like *histidine decarboxylase (Hdc); galactosidase beta 1 like 3 (Glb1l3),* which is associated with Leber’s congenital amaurosis; as well as *serine protease 56 (Prss56)*, which was reported to be involved in eye development, were significantly downregulated. Genes controlling scar formation, such as *fibrinogen-like 2 (Fgl2)* as well as apoptosis, e.g., *caspase1 (Casp1)* or *Bcl2-interacting killer (apoptosis-inducing) (Bik)*, were significantly higher expressed in VPP retinae. Furthermore, we found upregulation of quite a considerable number of genes associated with inflammatory or immune response functions such as *C-X-C motif chemokine ligand 13 (Cxcl13)*, *glial fibrillary acidic protein (Gfap)*, *T-cell receptor T3 gamma chain (Cd3g)*, *chemokine (C-C motif) ligand 5 (Ccl5),* and *C-C motif chemokine ligand 2 (Ccl2)*, as well as factors associated with the complement cascade like *complement component factor i (Cfi)*, *complement factor C4B*, and the *Serping1* gene.

Gene ontology enrichment showed, among others, involvement of TGF-β regulated extracellular matrix organization, response to cytokine stimuli, and disturbed cellular homeostasis in VPP retinae (Table 1). Photoreceptor loss was indicated by a lower expression level of rhodopsin signaling pathway components (Table 1). Moreover, we performed weighted gene correlation network analysis (WGCNA) to identify genotype-specific patterns of dysregulation, upstream regulators, and involved signaling pathways. WGCNA clusters co-regulated genes into modules based on their similarity of expression. As this approach does not rely on the traditional dysregulation analysis and the problem of correction for multiple comparisons, more subtle changes and patterns can be identified. In addition, biological key players, e.g., regulatory proteins driving a certain pathway, for a given module can be found by the intra-module analysis. The topology overlay matrix, which represents the co-regulation of expression, for VPP and control animals, as well as the identified modules (clusters of co-regulated genes as shown by their colors in Figure 1B) and their correlation of expression with the genotype, are illustrated in Figure 1B. The analysis identified six significantly associated modules (three positively correlated with the genotype, i.e., higher expressed in the VPP animals (Pos1, 2, 3) and three negatively correlated, i.e., lower expressed in the VPP animals (Neg1, 2, 3) (Figure 1D,E and Supplementary Figure S2A–D)). The Pos1 module was significantly enriched for genes involved in cellular transport and differentiation (Figure 1D and Table 2). In the Pos2 module, we found significant clustering of genes regulating necroptosis, protein transport, organelle organization, cellular homeostasis, and the ribosome (Supplementary Figure S2A and Table 2). The Pos3 module (Supplementary Figure S2B and Table 2) showed enrichment for bone morphogenetic protein (BMP) signaling pathway components. In the Neg1 module, we observed significant clustering of genes regulating cellular component organization and protein kinase activity (Figure 1E and Table 2). The Neg2 module (Supplementary Figure S2C and Table 2) was enriched for genes involved in nucleotide homeostasis and the Neg3 module showed significant enrichment for genes encoding for ribosomal proteins and transport proteins (Supplementary Figure S2D and Table 2).

### 2.3. Dysregulation of Potentially Neuroprotective Pathways in VPP Retinae: VEGF-, TGF-β-, and G-protein Mediated Signaling 

As a follow up to our previously published studies [10,13,14] on the neuroprotective properties of signaling pathways such as transforming growth factor (TGF)-β signaling, G-protein activated signaling, and vascular endothelial growth factor (VEGF) mediated signaling, we investigated their potential regulation in the VPP model. Quite intriguingly, our RNAseq data analysis (Supplementary Table S2) showed a significant upregulation of genes encoding for components of the G-protein activated signaling family. Here, we particularly focused on endothelin signaling, as our group and others recently showed that *endothelin 2 (Edn2)* and *endothelin receptor b (Ednrb)* are upregulated following photoreceptor damage [10,11,12,15]. In accordance, the RNAseq data (Supplementary Table S2) of the VPP retinae showed a significant increase in *Ednrb* (1.28-fold, *p_adj_* = 0.0074) and *Edn2* (22.12-fold, *p_adj_* = 8.68 × 10^−87^) expression. We furthermore observed an upregulation of factors involved in TGF–β signaling (e.g., *Tgf-β receptor type 1 (Tgfbr1)*: 1.15-fold, *p_adj_* = 0.013; *Tgfbr2*: 2.23-fold, *p_adj_* = 2.18 × 0^−23^; *Tgf-β1*: 2.24, *p_adj_* = 1.24 × 10^−12^; *Tgf-β2*: 1.51-fold, *p_adj_* = 2.20 × 10^−20^). Of note, *Tgf-β2* was additionally identified as one of the hub genes in the Pos1 module. Vascular endothelial growth factor (VEGF) receptor signaling pathway was identified in the gene ontology enrichment analysis of the significantly upregulated genes and the RNAseq data (Supplementary Table S2) showed an increased expression of *Vegfr1 (Flt1)*: 1.25-fold, *p_adj_* = 4.75 × 10^−7^, *Vegfr2 (Kdr)*: 2.14-fold, *p_adj_* = 1.40 × 10^−41^; *Vegfb*: 1.20-fold, *p_adj_* = 0.00013; and *Vegfc*: 1.46-fold, *p_adj_* = 9.31 × 10^−5^ (Supplementary Table S2).

**Figure 2:**
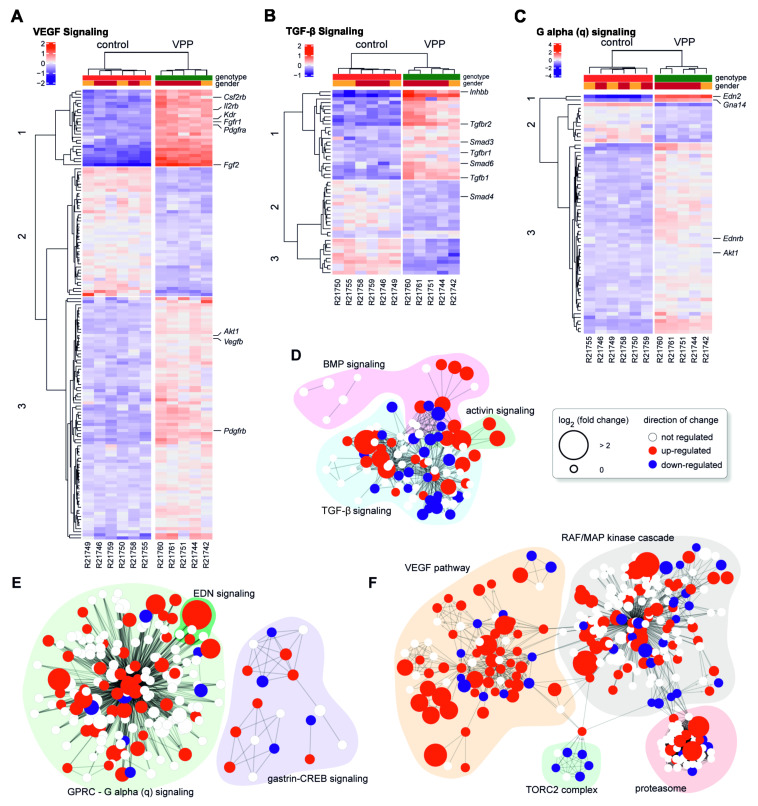
**Pathway analyses of transcriptomic changes in VPP mice.** (**A**–**C**) Heatmaps showing the significantly dysregulated genes in the Reactome pathways VEGF signaling (**A**), TGF-β signaling (**B**), and G alpha (q) signaling (**C**). For each heatmap, the genotypes separate perfectly, as indicated by the unsupervised clustering above the heatmaps. Colors (red: upregulated, blue: downregulated) represent the deviation of the mean expression for each gene, independent of genotype. K-mer analysis into three groups revealed clusters of tightly co-regulated genes. Some interesting genes (e.g., neuroprotective or immune modulating function, directly involved in the intracellular signaling) are highlighted on the right. To further visualize sub-groups of pathways that were dysregulated, we converted the Reactome pathways into functional interaction networks. For each network, genes were colored according to their dysregulation state: white—not significantly dysregulated; red—significantly upregulated; and blue—significantly downregulated. The size of the nodes corresponds to the log2-fold change of regulation. The network for TGF-beta signaling is shown in (**D**), G alpha (q) signaling is shown in (**E**), and VEGF signaling is shown in (**F**); R21742-61 = RNAseq sample number.

Thus, we aimed to investigate the impact of these signaling pathways (TGF-β-, G-protein activated-, and VEGF- signaling) in the VPP model in detail. Unsupervised hierarchical clustering of the samples that we generated on basis of the Reactome pathway database [16] for the VEGF (Figure 2A), TGF-β (Figure 2B) and G-protein mediated signaling pathways (Figure 2C) demonstrated a perfect separation of the genotypes, highlighting the dysregulation of these pathways in the VPP animals. Furthermore, k-mer analysis (three k-mer groups indicated by numbers on the left side of each heatmap) showed clusters of tightly co-regulated genes. We highlighted some genes of particular interest (e.g., which are known to be involved in neuroprotective or immune modulating processes or to be involved in regulatory functions) in each pathway on the right side of each heatmap. The heatmaps including the full labelling are shown in Supplementary Figure S3. To further analyze sub-groups of dysregulated pathways, we transformed the Reactome pathways into functional interaction networks (Figure 2D–F). We colored the genes of each network according to their dysregulation: white indicates no significant regulation, red genes were up- and blue genes were significantly down-regulated, respectively. The size of each node corresponds to the log2-fold change of regulation. The fully labeled networks are shown in Supplementary Figure S4. This analysis identified distinct sub-clusters of dysregulated genes, e.g., *endothelin 2 (Edn2)* and *endothelin receptor b (Ednrb)* in the G-protein activated signaling pathway network (Figure 2E and Supplementary Figure S4C) or *Tgfbr2* in the TGF-β family signaling network (Figure 2D and Supplementary Figure S4B) and *Vegfr2/kinase insert domain receptor (Kdr)* in the VEGF signaling network (Figure 2F and Supplementary Figure S4A). 

**Figure 3:**
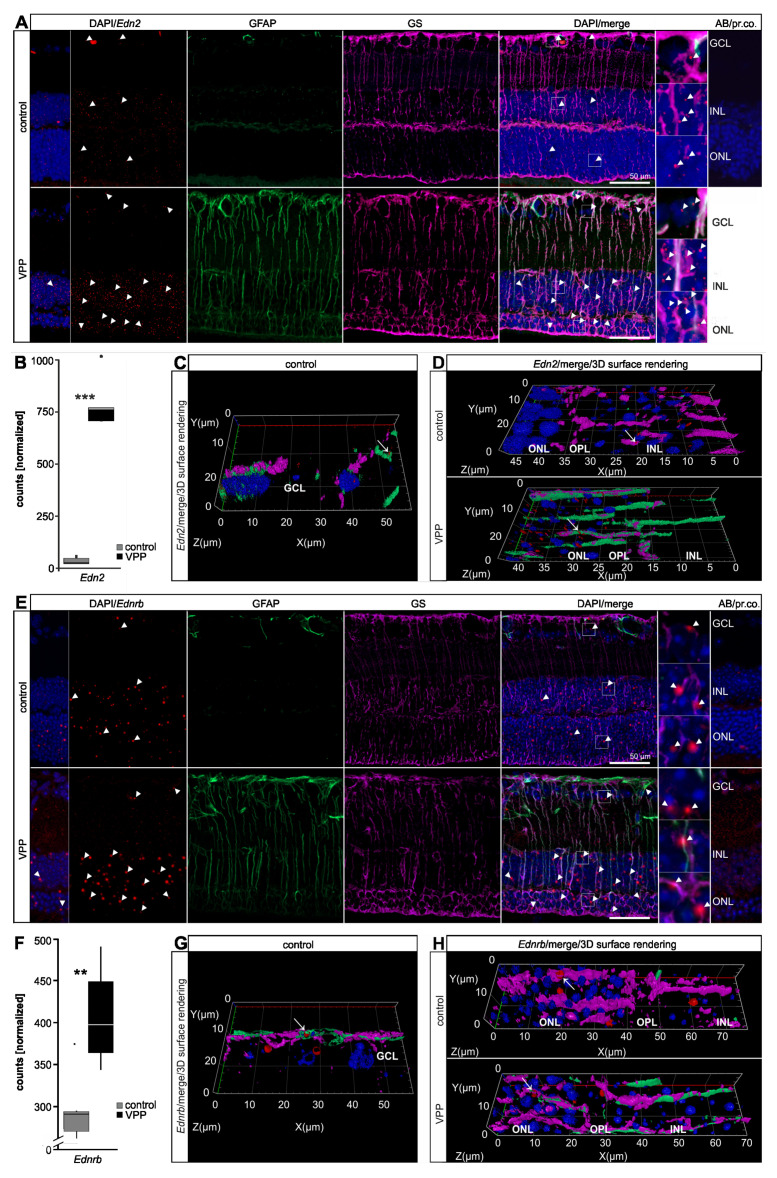
Upregulation of endothelin signaling in VPP mice. (**A**) In situ hybridization for *Edn2* (red, arrowheads) and GFAP (green)/GS (purple) immunofluorescence co-labeling in the retinae of three-month-old animals. Nuclei were DAPI-stained (blue). In the VPP retina, the number of the *Edn2* signals (red, arrowheads) was increased and the Müller cells were GFAP/GS-positive. The boxed areas in the merge image are shown in high resolution on the right. (**B**) Boxplots showing the extracted *Edn2* expression data from the RNAseq as normalized counts for control and VPP genotypes. Control *n* = 6; VPP *n* = 5; *** *p_adj_* = 8.68 × 10^−87^. (**C**,**D**) Higher magnification of the GCL (**C**) and ONL/OPL/INL region (**D**) depicted as 3D reconstruction (*Edn2*/merge/3D surface rendering). (**C**) *Edn2* signals (red, arrow) partly overlapped with GFAP (green)-positive astrocytes. (**D**) *Edn2* punctae (red, arrow) overlapped to some extent with GS (purple)-positive resting (control animal, arrow) and GFAP (green)/GS (purple)-positive reactive (VPP animal, arrow) Müller cells. (E) In situ hybridization for *Ednrb* (red, arrowheads) and GFAP (green)/GS (purple) immunofluorescence co-labeling in the retinae of three-month-old animals. Nuclei were DAPI-stained (blue). In the VPP retina, the Müller cells were GFAP/GS-positive. The boxed areas in the merge image are shown in high resolution on the right. (**F**) Boxplots showing the extracted *Ednrb* expression data from the RNAseq as normalized counts for control and VPP genotypes. Control *n* = 6; VPP *n* = 5; ** *p_adj_* = 0.0074. (**G**,**H**) Higher magnification of the GCL (**G**) and ONL/OPL/INL region (**H**) depicted as a 3D reconstruction (*Ednrb*/merge/3D surface rendering). (**G**) *Ednrb* signals (red, arrow) partly overlapped with GFAP (green)-positive astrocytes. (**H**) *Ednrb* punctae (red, arrow) overlapped to some extent with GS (purple)-positive resting (control animal, arrow) and GFAP (green)/GS (purple)-positive reactive (VPP animal, arrow) Müller cells. *Edn2* = *endothelin2*; *Ednrb* = *endothelin receptor type B*; GCL = ganglion cell layer; INL = inner nuclear layer; OPL = outer plexiform layer; ONL = outer nuclear layer; GFAP = glial fibrillary acidic protein; GS = glutamine synthetase; AB/pr. co. = antibody/probe control.

In addition to the quantitative information from the RNAseq data derived from whole retinae, and to further validate our results, we next performed mRNA in situ hybridization and/or immunofluorescence staining to identify cell types expressing transcripts of interest. Using a specific probe against *Edn2,* we detected *Edn2* most prominently in the ONL in the control retina. However, we also observed distinct *Edn2* signals in the INL and some rather sparse signals in the GCL (Figure 3A). VPP retinae showed significantly higher *Edn2* expression in the RNAseq data (22.12-fold, *p_adj_* = 8.68 × 10^−87^; Figure 3B, Supplementary Table S2, Supplementary Figure S1G) and accordingly showed a marked increase of *Edn2* signals in particular in the degenerating ONL and in the INL, but the signals in the GCL remained sparse (Figure 3A). To identify specific cell types expressing *Edn2*, we combined in situ hybridization with immunofluorescence staining. We used glial fibrillary acidic protein (GFAP) as a marker for astrocytes and reactive Müller cells. GFAP is a major intermediate filament particularly expressed in astrocytes [17] and upregulated in response to retinal trauma in astrocytes and Müller cells [17,18]. Moreover, immunofluorescence staining for glutamine synthetase (GS) was used to label Müller cells [19]. When performing GFAP/GS/*Edn2* co-labelling, we observed the characteristic GFAP staining pattern for astrocytes in the nerve fiber layer for both groups (Supplementary Figure S5A,B). Consistent with the observed upregulation of *Gfap* in VPP retinae as determined by RNAseq analysis (17.05-fold, *p_adj_* = 3.87 × 10^−78^), immunofluorescence staining showed an increased GFAP signal intensity, indicating an enhanced protein expression, in the nerve fiber layer and an additional stripe-like staining pattern stretching through the retina, which represents the characteristic morphological appearance of reactive Müller cells. Of note, unlike apoptosis and ONL thinning (Supplementary Figure S1A,D,E), which were both more prominent in the central retina, we could not detect a difference in GFAP reactivity between the central (Supplementary Figure S5A,B) and the peripheral (Supplementary Figure S5A,C) parts of the retina (VPP animals (*n* = 3): GFAP-intensity central retina: 26,653.88 mean gray value/mm²; GFAP-intensity peripheral retina 50,308.26 mean gray value/mm², *p* = 0.10). In the 3D reconstruction of *Edn2*/GFAP/GS co-labelled sections, we observed some *Edn2* signals in GFAP-positive astrocytes (Figure 3C and Supplementary Figure S6C) and in GS-positive resting and GFAP/GS-positive reactive Müller cells, respectively (Figure 3D and Supplementary Figure S6D). However, we observed the majority of the *Edn2* signals in neurons of the INL and ONL (Figure 3A).

*Ednrb* mRNA in situ hybridization showed specific signals in the INL and ONL and some defined signals in the GCL in control retinae (Figure 3E). We furthermore observed some *Ednrb* expression in GFAP-positive astrocytes (Figure 3G and Supplementary Figure S6E) and in GS-positive resting Müller cells (Figure 3H and Supplementary Figure S6F). In VPP retinae, *Ednrb* was significantly upregulated (1.29-fold, *p_adj_* = 0.007; Figure 3F) in the RNAseq data (Supplementary Table S2). In the 3D reconstruction of the *Ednrb*/GFAP/GS labelling, we detected pronounced *Ednrb* signals in the INL and ONL that overlapped to some extent with GFAP-/GS-positive reactive Müller cells (Figure 3E,H and Supplementary Figure S6F). Yet, *Ednrb* signals were visible in the INL and ONL that did not overlap with GFAP/ GS, indicating its further expression in neuronal cells.

**Figure 4:**
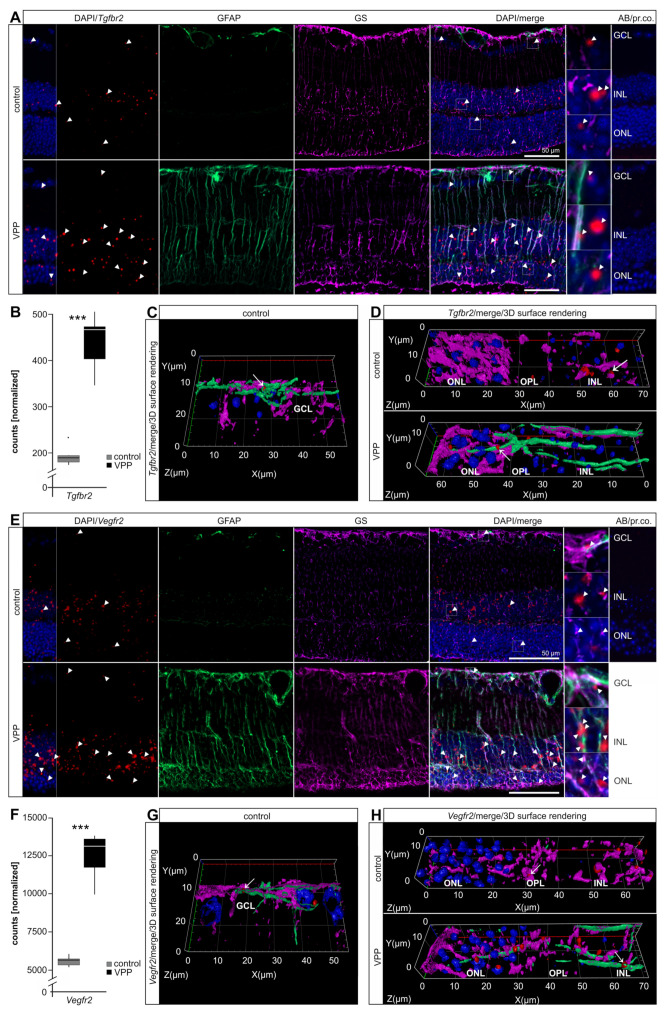
Upregulation of TGFβ- and VEGF-signaling in VPP mice. (**A**) In situ hybridization for *Tgfbr2* (red, arrowheads) and GFAP (green)/GS (purple) immunofluorescence co-labeling in the retinae of three-month-old animals. Nuclei were DAPI-stained (blue). In the VPP retina, the number of *Tgfbr2* signals (red, arrowheads) in the ONL and INL was increased and the Müller cells were GFAP/GS-positive. The boxed areas in the merge image are shown in high resolution on the right. (**B**) Boxplots showing the extracted *Tgfbr2* expression data from the RNAseq as normalized counts for control and VPP genotypes. Control *n* = 6; VPP *n* = 5; *** *p_adj_* = 2.18 × 10^−23^. (**C**,**D**) Higher magnification of the GCL (**C**) and ONL/OPL/INL region (**D**) depicted as 3D reconstruction (*Tgfbr2*/merge/3D surface rendering). (**C**) *Tgfbr2* signals (red, arrow) showed only scattered co-labeling with GFAP (green)-positive astrocytes. (**D**) *Tgfbr2* punctae (red, arrow) partly associated with GS (purple)-positive resting (control animal, arrow) and GFAP (green)/ GS (purple)-positive reactive (VPP animal, arrow) Müller cells. (**E**) In situ hybridization for *Vegfr2/Kdr* (red, arrowheads) and GFAP (green)/GS (purple) immunofluorescence co-labeling in the retinae of three-month-old animals. Nuclei were DAPI-stained (blue). The number of *Vegfr2* signals (red, arrowheads) was increased in the VPP retina and the Müller cells were GFAP/GS-positive. The boxed areas in the merge image are shown in high resolution on the right. (**F**) Boxplots showing the extracted *Vegfr2/Kdr* expression data from the RNAseq as normalized counts for control and VPP genotypes. Control *n* = 6; VPP *n* = 5; *** *p*_adj_ = 1.40 × 10^−41^. (**G**,**H**) Higher magnification of the GCL (**G**) and ONL/OPL/INL region (**H**) depicted as a 3D reconstruction (*Vegfr2*/merge/3D surface rendering). (**G**) *Vegfr2* signals (arrow) showed some co-labeling with GFAP (green)-positive astrocytes. (**H**) *Vegfr2* signals partly overlapped with GS (purple)-positive resting (control animal, arrow) and GFAP (green)/GS (purple)-positive reactive (VPP animal, arrow) Müller cells. *Tgfbr2* = *transforming growth factor beta receptor type 2*; *Vegfr2* = *vascular endothelial growth factor receptor 2*; GCL = ganglion cell layer; INL = inner nuclear layer; OPL = outer plexiform layer; ONL = outer nuclear layer; AB/pr. co. = antibody/probe control.

*Tgfbr2* was significantly upregulated (2.23-fold, *p_adj_* = 2.18 × 10^−23^; Figure 4B) in VPP retinae in the RNAseq data (Supplementary Table S2). To further supplement the quantitative information and potentially identify cell types in which it is upregulated, we performed *Tgfbr2* in situ hybridization. Control retinae showed distinct signals in the INL and ONL and some scattered punctae in the GCL (Figure 4A and Supplementary Figure S6G,H). In VPP retinae, the number of *Tgfbr2* punctae increased in the INL and ONL. 3D reconstruction of immunofluorescence co-labelling confirmed its expression in only some isolated GFAP-positive astrocytes (Figure 4C and Supplementary Figure S6G) and its association with resting, GS-positive, and reactive GFAP-/GS-positive Müller cells, respectively (Figure 4D and Supplementary Figure S6H). Yet, we also observed *Tgfbr2* in situ hybridization in the neuronal cell layers of the retina, in particular in the INL and ONL, that did not overlap with GFAP-/GS-positive Müller cells, indicating additional expression in neuronal cells (Figure 4A).

*Vegfr2* mRNA in situ hybridization in control retinae showed numerous signals in the INL that partly overlapped with GS-positive resting Müller cells (Figure 4E and Supplementary Figure S6J). Moreover, we detected *Vegfr2* mRNA in the ONL and isolated signals in the GCL that overlapped to some extent with GFAP-positive astrocytes (Figure 4E,G and Supplementary Figure S6I). Our RNAseq analysis showed *Vegfr2* to be significantly upregulated in VPP retinae (2.00-fold, *p_adj_* = 1.40 × 10^−41^; Figure 4F; Supplementary Table S2) and, accordingly, *Vegfr2* in situ hybridization showed an increase in expression in the INL and ONL (Figure 4E). 3D reconstruction of co-labelling showed its association with and expression in GFAP-/GS-positive reactive Müller cells (Figure 4H and Supplementary Figure S6J). Moreover, we detected *Vegfr2* signals in the neuronal layers of the retina, again in particular in the INL and ONL, that did not overlap with GFAP-/GS-positive Müller cells, indicating additional expression in neuronal cells (Figure 4E).

### 2.4. The Glial Response to Photoreceptor Degeneration in VPP Mice

**Figure 5:**
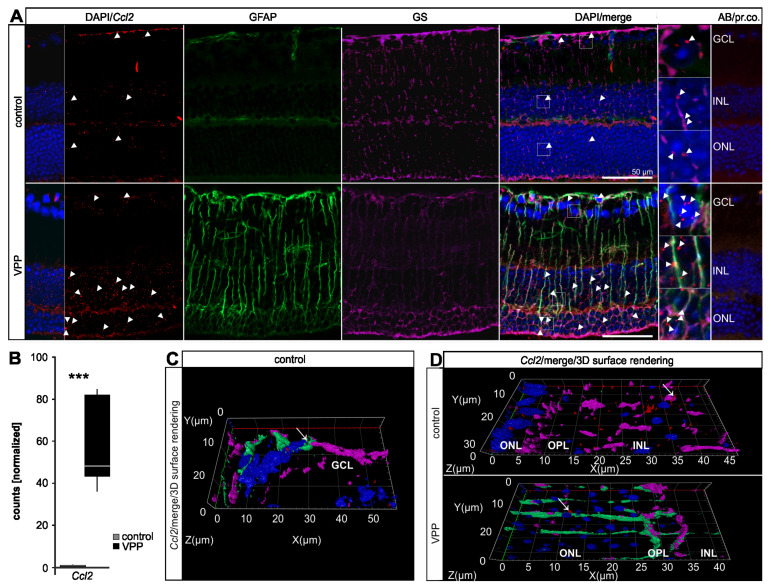
Upregulation of *Ccl2* in VPP mice. (**A**) In situ hybridization for *Ccl2* (red) and GFAP (green)/GS (purple) immunofluorescence co-labeling in the retinae of three-month-old animals. Nuclei were DAPI-stained (blue). In the control retina, a rather discrete *Ccl2* signal (arrowheads) was visible. In the VPP retina, the number of the *Ccl2* punctae increased (arrowheads) and the Müller cells were GFAP/GS-positive. The boxed areas in the merge image are shown in high resolution on the right. (**B**) Boxplots showing the extracted *Ccl2* expression data from the RNAseq as normalized counts for control and VPP genotypes. Control *n* = 6; VPP *n* = 5; *** *p_adj_* = 8.05 × 10^−14^. (**C**,**D**) Higher magnification of the GCL (**C**) and ONL/OPL/INL region (**D**) depicted as a 3D reconstruction (*Ccl2*/merge/3D surface rendering). (**C**) One of the few overlaps of the *Ccl2* signals (red, arrow) with GFAP (green)-positive astrocytes. (**D**) *Ccl2* punctae (red, arrow) partially overlapped with GS (purple)-positive resting (control animal, arrow) and GFAP (green)/ GS (purple)-positive reactive (VPP animal, arrow) Müller cells. GCL = ganglion cell layer; INL = inner nuclear layer; OPL = outer plexiform layer; ONL = outer nuclear layer; Ccl2 = CC-chemokine ligand 2; GS = glutamine synthetase; AB/pr. co. = antibody/probe control; AU = arbitrary unit.

As we observed a significant upregulation of several genes modulating glial reactivity (e.g., *glial fibrillary acidic protein (Gfap), serine protease inhibitor A3N (Serpina3n), lipocalin 2 (Lcn2*), we performed qPCR analyses on retinal samples to determine the relative *Gfap* expression levels (control: 1.00 ± 0.19, VPP: 8.89 ± 0.63, *p* < 0.001) as well as the expression levels of the microglia/macrophage marker *ionized calcium-binding adapter molecule 1* (*Iba1*, control: 1.00 ± 0.11, VPP: 6.56 ± 1.03, *p* = 0.015) and the *chemokine (C-C motif) ligand 2* (*Ccl2*, control: 1.00 ± 0.11, VPP: 68.74 ± 5.11, *p* < 0.001), the latter being reported to stimulate the migration and reactivity of microglia cells [20,21] (Supplementary Figure S5D). In accordance, our RNAseq data showed increased *Iba1* (5.51-fold, *p_adj_* = 1.12 × 10^−23^ expression levels), which we further validated using an anti-IBA1 labeling to visualize myeloid cells, e.g., microglia and recruited macrophages, in the retinae of control and VPP animals (Supplementary Figure S5E,F). In controls, we observed ramified IBA1-positive cells in their typical localization in the nerve fiber layer and the inner (IPL) and outer plexiform layers (OPL). In contrast, in VPP retinae, IBA1-positive cells changed their shape from ramified microglia towards amoeboid, reactive microglia in particular in the OPL, and thus in very close association with the degenerating photoreceptors. Moreover, we observed an accumulation of amoeboid-shaped, IBA1-positive cells in the sub-neuroretinal space in close proximity to the retinal pigment epithelium (RPE) (Supplementary Figure S5E,F). Taken together, these results showed a pronounced reactivity of macro- and microglial cells in response to photoreceptor degeneration. To identify the origin of the significantly elevated *Ccl2* expression in VPP retinae and to supplement the quantitative information from the RNAseq (*Ccl2*: 67.51-fold increase, *p_adj_* = 8.05 × 10^−14^; Figure 5B) and qPCR data (Supplementary Figure S5D), we performed *Ccl2* mRNA in situ hybridization on retinal sections combined with immunofluorescence co-labeling of glial cells. In control retinae, we observed a rather low number of *Ccl2* punctae in the inner nuclear layer (INL) and the ONL, and a few signals in the retinal ganglion cell layer (GCL) (Figure 5A and Supplementary Figure S6A,B). The number of *Ccl2* punctae was markedly increased in the INL and ONL of the VPP retinae (Figure 5A). When performing 3D reconstruction of *Ccl2*/GFAP/GS co-labelled sections, we observed sparse overlap of *Ccl2* in GFAP-positive astrocytes (Figure 5C and Supplementary Figure S6A) and more frequent overlap in GS-positive resting and in GFAP-/GS-positive reactive Müller cells (Figure 5D and Supplementary Figure S6B). However, we also detected *Ccl2* mRNA expression in cells other than Müller glia and astrocytes in the neuronal layers of the retina (GCL, INL, and ONL), pointing towards additional expression in retinal neurons (Figure 5A).

## 3. Discussion

The present data confirm that the VPP model displays the major phenotypic characteristics of the human disease retinitis pigmentosa. Briefly, we demonstrate, in comprehensive transcriptome-wide analyses of retinae from three-month-old VPP mice, (1) an extensive dysregulation of genes modulating apoptosis, processes in scar formation, and components of the (ocular) immune system or response, respectively; (2) a strong genotype-dependent clustering of genes regulating the VEGF, TGF-β, and G-protein activated signaling pathway; (3) the expression of regulatory genes in neurons as well as resting and reactive glia cells; and (4) a dysregulation of extracellular matrix organization and cellular homeostasis in WGCNA analyses.

### 3.1. The Transcriptional Response to Photoreceptor Degeneration Leads to Increased Expression of Genes Regulating Inflammatory or Immune Response Functions

Neuroinflammation is a common hallmark of the pathogenesis of neurodegenerative diseases like Alzheimer’s, Parkinson’s, multiple sclerosis, or retinal degenerations [22,23,24]. Following a neurotoxic event, neuronal stress signals mediate reactivity of microglial cells, leading to their proliferation, migration, and the secretion of specific cytokines and chemokines that can exert neurotoxic or neuroprotective effects [23,25]. Sustained reactivity of microglia promotes chronic inflammation and may cause irreversible neuronal cell death [23,26,27]. Thus, the accumulation of reactive IBA1-positive cells in the OPL and in the sub-neuroretinal space in VPP retinae strongly indicates an ongoing neuroinflammatory process. Moreover, in the top 30 dysregulated genes, we found a considerable number of genes associated with inflammatory or immune response functions, respectively. Gene ontology enrichment analyses also pointed towards an upregulation of the cellular response to cytokine stimuli, again indicating an ongoing neuroinflammation. These findings are in accordance with previously published data, which describe upregulation of factors like *Lif, Ccl2 (Mcp-1)*, *Ccl28, interleukin-1 (Il-1), complement component 1q (C1q)*, and *complement factor H (CFH)* in retinae of genetic mouse models of RP [11,28,29,30,31,32,33]. Quite intriguingly, microarray data from the retina of one RP patient carrying two mutations in the *ABCA4* gene showed, among others, an increased expression of complement system genes (*complement factor B, complement C2*), several cytokines, and cytokine receptors (*IL- 6*, *CXCL10*, *CXCL2*), respectively, indicating a neuroinflammatory process in human RP [24]. We as well as others [20,21] have shown that *Ccl2* is expressed in Müller cells and photoreceptors in the healthy retina and upregulated upon retinal damage, contributing to the recruitment of microglia/infiltrative macrophages [21,34]. However, conflicting data exist concerning the exact role of Ccl2 in the context of neurodegeneration. Recently, Joly and colleagues showed that the retinal morphology of double mutant mice expressing the VPP transgene on a *Ccl2* null background (VPP; Ccl2^−/−^) did not differ from that of transgene VPP mice on a wildtype background [11]. In contrast, Rutar and colleagues demonstrated that siRNA-mediated knock down of *Ccl2* resulted in a significantly lower number of apoptotic photoreceptors in rats after light-induced photoreceptor degeneration and [21,34]. Based on our data, we hypothesize that, in VPP retinae, the elevated *Ccl2* expression in Müller cells and in the ONL contributes to the attraction/migration and reactivity of microglial cells in this particular region. In our comprehensive analyses, we furthermore detected a considerable number of genes encoding for components of the complement system, which is part of the innate immune system. Various complement factors have been reported to be upregulated in retinae of human patients suffering from RP or in retinae of mice following genetically or light-induced photoreceptor degeneration in mice [30,31,32,35,36,37,38]. Activation of the complement system promotes microglia/infiltrative macrophages migration and eventually complement activated lysis [31,35]. Still, conflicting data exist regarding the exact role of complement system activation and its impact on photoreceptor degeneration. Mice with a deficiency in complement factor D are protected from light-induced photoreceptor degeneration [30], indicating a detrimental role for photoreceptor survival, but the deficiency in complement component 3 (C3) or complement receptor 3 (CR3) in a genetic mouse model of photoreceptor degeneration increases microglia-mediated neurotoxicity to photoreceptors [38]. Thus, the detailed function of the complement system and its specific role in microglia and Müller cells and its contribution to photoreceptor degeneration has yet to be elucidated. Nevertheless, based on our transcriptome-wide data, we conclude that the significant upregulation of genes associated with inflammatory or immune response functions leads to neuroinflammation in VPP retinae, potentially contributing to the degeneration of photoreceptors.

### 3.2. The Transcriptional Response to Photoreceptor Degeneration Leads to the Upregulation of Neuroprotective Factors and Pathways 

We additionally analyzed the impact of the VPP model on neuroprotective pathways like TGF-β, G-protein, and VEGF signaling [39]. Recently, our group and others showed that, in response to retinal injury, endothelin 2 *(Edn2)* is expressed by photoreceptors concomitant with elevated expression of *endothelin receptor b* (*Ednrb)* and *Gfap*, the latter indicating the reactivity of Müller cells, and an increased expression of *Lif* and *Fgf2* [10,11,12,15,40]. Our RNAseq data, in situ hybridizations, immunofluorescence staining, and qPCR analyses confirmed this observation for the VPP model of retinal degeneration. It is of interest to note that our co-labelling experiments showed expression not only of *Ednrb*, but also of *Tgfbr2* and *Vegfr2* in resting and reactive Müller cells, clearly indicating the close interplay of neuronal and glial cells. Furthermore, we previously showed that *Edn1, Edn2, Ednra,* and *Ednrb* are significantly upregulated following induced ocular traumata [15]. Yet, in VPP retinae, only *Edn2* and *Ednrb* were upregulated. These inconsistent results might well be explained by the different activation patterns of signaling pathways depending on the actual cause of cell death, i.e., light-induced versus genetically-induced cell death [28].

### 3.3. The Transcriptional Response to Photoreceptor Degeneration Leads to Expression of Pro-Apoptotic Factors and Extracellular Matrix Organization

Our transcriptome analyses also showed upregulation of factors associated with apoptosis (*Casp1, Bik*) and scar formation such as *fibrinogen like 2 (Fgl2)* and *Tgf-β1.* A well-described characteristic of TGF-β signaling is its contribution to wound healing, tissue fibrosis, and scar formation [41,42]. Accordingly, “TGF-β regulated organization of extracellular matrix” was a major hit in our gene ontology analysis and might well be the result of a healing response following photoreceptor degeneration in VPP retinae. We furthermore identified the isoform *Tgf-β2* as one of the central hub genes in the WGCNA module Pos1. Gene ontology analysis of the Pos1 module showed enrichment for genes involved in cell differentiation. TGF-β signaling modulates manifold processes, e.g., the regulation of early development, cell-cycle control, and cell differentiation [43,44,45]. Moreover, we recently showed that the deletion of TGF-β signaling results in the development of retinal microaneurysms and choroidal neovascularization [13,46], clearly emphasizing its potential to regulate angiogenic processes. Furthermore, TGF-β signaling contributes to extracellular matrix reorganization in ocular diseases such as primary open angle glaucoma [47,48,49]. However, AAV-mediated delivery of TGF-β1 rescued degenerating cones in mouse models of RP [50] and TGF-β signaling protects retinal neurons from programmed cell death during retinal development [14], thus highlighting its neuroprotective properties [51].

The observed gliosis of astrocytes and Müller cells, as indicated, e.g., by elevated *Gfap* expression levels and the characteristic stripe-like staining pattern of GFAP in retinal sections, is a typical reaction of neuronal tissue to various neurotoxic insults [18,52] and eventually results in a glial scar. Quite intriguingly, recently published data suggest a major and interactive role of glial cells such as astrocytes contributing to neurodegeneration and their cell-specific targeting resulted in accelerated functional recovery compared with untreated animals [53,54].

The identified dysregulation of genes involved in neuroinflammation, neuroprotection, apoptosis, scar formation, and wound healing and the corresponding WGCNA data are not only of interest for researchers working on retina, but might well be of interest for scientists working with other neuronal tissues.

In the future, using material from mouse models of RP or humanized 3D culture models (retinal organoids) combined with advanced transcriptomic techniques such as imaging-based in situ cell-type identification/mapping combined with single-cell RNA-sequencing will allow to create a molecularly annotated and spatially dedicated cell atlas of transcriptional changes related to RP [55,56]. Moreover, the use of high-throughput screening and computer-aided drug design can provide novel insights with the overall aim to find new treatment options for neurodegenerative diseases such as RP [57,58].

## 4. Material and Methods

### 4.1. Mice

The mice were on a 129 SV background and kept in a 12 h light/dark cycle. Mice of both sexes were used for the experiments. Mice, carrying two floxed *Tgfbr2* alleles at chromosome 9 [59], thus representing functional wildtype mice, were crossbred with hemizygous VPP mice [6]. The resulting offspring expressed either wildtype rhodopsin (named as control mice = VPP negative animals) or the VPP transgene (referred to as VPP mice), a rhodopsin mutant with point mutations at positions V20G, P27L, and P23H, in addition to wildtype rhodopsin. The VPP mutation results in a progressive retinal neurodegeneration [6]. For genotyping, the following primers were used: 5’-agactgacatggggaggaattcccaga-3´ (sense) and 5´-gagctgctcgaagtgactccgacc-3´ (antisense). The thermal cycle protocol was denaturation at 94 °C for 30 s, annealing at 68 °C for 45 s, and elongation at 72 °C for 45 s for 35 cycles.

### 4.2. Microscopy and Morphometric Analyses (Spider Diagram)

The enucleated eyes were fixed for 24 h in 2.5% paraformaldehyde (PFA)/2.5% glutaraldehyde in sodium cacodylate buffer and processed as described previously [60]. Then, 1 µm thick semithin meridional sections were cut and stained according to Richardson [61]. The sections were analyzed on an Axio Imager Z1 microscope (Carl Zeiss, Jena, Germany) using Zeiss Zen software (Carl Zeiss, Jena, Germany). The thickness of the outer nuclear layer (ONL) was measured and the mean values were plotted a as spider diagram, as described previously in [13,40]. It is of interest to note that there was no sex-specific difference in the ONL thickness of VPP (male (*n* = 3) versus female (*n* = 3)) and control (male (*n* = 3) versus female (*n* = 3)) animals.

### 4.3. Apoptosis: TdT-Mediated dUTP-Biotin Nick End Labeling (TUNEL) 

TUNEL (DeadEnd Fluorometric TUNEL, Promega, Madison, WI, USA) was used to label apoptotic cells in one-month-old animals, following our previously published protocol [10,14]. The sections were analyzed on an Axio Imager Z1 microscope (Carl Zeiss, Jena, Germany) using Zeiss Zen software (Carl Zeiss, Jena, Germany). Eyes of eleven animals (VPP: *n* = 11, control: *n* = 11) were included in the morphometric analyses and their TUNEL-positive cells were counted and normalized to the area of the ONL [mm^2^]. There was no sex-specific difference in the number of TUNEL positive cells between VPP (male (*n* = 6) versus female (*n* = 5)) animals.

### 4.4. Immunofluorescence and RNA/Basescope^®^ In Situ Hybridization

Eyes were fixed for 4 h in 4% PFA, washed extensively in phosphate buffer (PB, 0.1 M, pH 7.4), and embedded in paraffin according to standard protocols. Paraffin sections (6 µm thick) were deparaffinized. Glial fibrillary acidic protein (GFAP) (Agilent Dako, Santa Clara, CA, USA), ionized calcium-binding adapter molecule 1 (IBA1) (Wako, Ōsaka, Japan), and glutamine synthetase (GS) (Millipore, Temecula, California, USA) immunofluorescent staining were performed as described in [13]. For in situ hybridization (ACD, Newark, USA), paraffin sections were pretreated with retrieval reagent and protease according to the user manual. RNAscope^®^ Fluorescent Multiplex Reagent Kit was used to hybridize *chemokine (C-C motif) ligand 2* (*Ccl2*) (ACD catalog number: 311791) and *Endothelin 2* (*Edn2*) (ACD catalog number: 418221) and BaseScope^TM^ Detection Reagent Kit v2—RED was used to label *Vegf receptor type 2* (*Vegfr2*) (ACD catalog number: 860711), *endothelin receptor type b* (*Ednrb*) (ACD catalog number: 706471), and *TGF-ß receptor type 2* (*Tgfbr2*) (ACD catalog number: 845871). The sections were analyzed on an Axio Imager Z1 microscope with the Apotome.2 function (Carl Zeiss, Jena, Germany) using Zeiss Zen software (Carl Zeiss, Jena, Germany).

To determine Müller cell reactivity of the peripheral and central part of the retina, we measured GFAP fluorescence intensity in the area of the peripheral and central 20% of the retina, which we defined as comparable to the measurements for the spider diagrams [13,40]. We thus used the feature “Intensity Mean Value” of the ZEN software (Carl Zeiss, Jena, Germany) to determine the mean grey value of GFAP and normalized the mean value of the intensity to the total retinal area from the GCL to the outer aspect of the ONL [mm^2^]. We found no difference in reactivity of Müller cells between the peripheral and central parts of the retinae in VPP animals (*n* = 3).

To study the co-localization of RNA/BaseScope and GFAP/GS, z-stacks (25–30 images, in total 5.5–7.0 µm thick) were analyzed, transformed into the ortho (orthogonal section) view, and reconstructed as a 3D image using the Zeiss Zen software and the 3Dxl rendering module with the surface function (Carl Zeiss, Jena, Germany).

### 4.5. RNA Isolation and Quantitative Real-Time RT-PCR (qPCR)

TriFast (Peqlab, Erlangen, Germany) was used to isolate total mRNA from retinal tissue and cDNA was synthesized using the iScript cDNA Synthesis Kit (Bio-Rad Laboratories, Inc., Hercules, CA, USA) following the manufacturer’s instructions. QPCR analyses were performed on a CFX Realtime PCR Detection System (Bio-Rad Laboratories, Inc.) and as previously described [46]. All oligonucleotides (Supplementary Table S1) were designed to span exon-intron boundaries and purchased from Invitrogen (Carlsbad, CA, USA). CFX Manager^TM^ Software and Excel were used to analyse relative mRNA expression levels according to the ∆∆C_T_-method [62]. The geometric mean value of the reference genes *ubiquitin (Ubc)* and *guanine nucleotide binding protein subunit beta2 like 1 (Gnb2l1)* was used for normalization. To perform RNA sequencing, total RNA of pooled retinae (right and left eye) was purified using the RNeasy Mini Kit by Qiagen (Venlo, The Netherlands).

### 4.6. RNA Sequencing

Library preparation and RNAseq were performed at the service facility ‘KFB—Center of Excellence for Fluorescent Bioanalytics’ (Regensburg, Germany. www.kfb-regensburg.de, accessed on 11 June 2021). Library preparation and RNAseq were carried out as described in the Illumina TruSeq Stranded mRNA Sample Preparation Guide, the Illumina NextSeq 500 System Guide (Illumina, Inc., San Diego, CA, USA), and the KAPA Library Quantification Kit—Illumina/ABI Prism User Guide (Kapa Biosystems, Inc., Woburn, MA, USA). In brief, 250 ng of total RNA was used for purifying the poly-A containing mRNA molecules using poly-T oligo-attached magnetic beads. Following purification, the mRNA was fragmented to an average insert size of 200–400 bases using divalent cations under elevated temperature (94 °C for 4 min). Next, the cleaved RNA fragments were reverse transcribed into first strand cDNA using reverse transcriptase and random hexamer primers. Actinomycin D was added to improve strand specificity by preventing spurious DNA-dependent synthesis. Blunt-ended second strand cDNA was synthesized using DNA Polymerase I, RNase H, and dUTP nucleotides. The incorporation of dUTP, in place of dTTP, quenched the second strand during the later PCR amplification, because the polymerase does not incorporate past this nucleotide. The resulting cDNA fragments were adenylated at the 3’ ends; the indexing adapters were ligated; and, subsequently, specific cDNA libraries were created by PCR enrichment. The libraries were quantified using the KAPA SYBR FAST ABI Prism Library Quantification Kit. Equimolar amounts of each library were sequenced on a NextSeq 500 instrument controlled by the NextSeq Control Software (NCS) v2.2.0, using a 75 Cycles High Output Kit with the single index, paired-end (PE) run parameters. Image analysis and base calling were done by the Real Time Analysis Software (RTA) v2.4.11. The resulting .bcl files were converted into .fastq files with the CASAVA Software v1.8.2.

### 4.7. Bioinformatics

For all samples, at least 30 million reads were analyzed. Fastq files were quality controlled with FastQC v0.11.5 [63]. All files passed quality control. The reads were aligned against Ensembl Mus musculus GRCm38 version 94 using STAR aligner v2.5.3a [64]. One sample (R21753) showed poor read alignments of less than 30% and was removed from further analyses. Reads were quantified using salmon v0.8.2 [65]. All subsequent analyses were conducted in R v3.5.1. Samples were screened for outliers using PCA and clustering analysis. One sample (R21741) was identified as an outlier and removed from further analyses. Thus, the final sample number was six control and five VPP retinae. Transcriptional dysregulation was computed using tximport v1.10.0 [66] and DESeq2 v1.22.1 [67] with genotype as the variable of interest and sex of the mice as a covariate and using ashr [68] as the fold change shrinkage estimator. The Benjamini–Hochberg procedure was used to correct for multiple comparisons (*p*-adjusted; *p*_adj_). For correlation network analysis, we used the normalized and variance stabilized counts from the DESeq2 analysis. Batch correction for sex was applied with limma v3.38.3 [69] keeping genotype as the variable of interest. The normalized, transformed, and batch corrected counts were used to construct a weighted gene correlation network using WGCNA v1.66 [70,71]. Heatmaps and k-mer analysis were carried out using ComplexHeatmap v2.3.2 [72]. Visualization was carried out using cytoscape v3.7.2 (http://cytoscape.org, accessed on 11 June 2021) with the Reactome FI app v7.2.1 [16]. Ontology analysis was carried out using the Enrichr website [73,74]. Scripts are available upon request.

### 4.8. Statistics

All results that are displayed in bar graphs are expressed as means ± SEM. Data were screened for outliers using the Grubb’s outlier test in graph pad prism. Comparisons between the means of two groups were made by a two-tailed Student’s t-test. *p* ≤ 0.05 was considered as statistically significant.

## 5. Conclusions

The parallel expression of VPP mutant and wildtype rhodopsin [6] results in a significant increase in apoptosis and thinning of the ONL to half of its thickness in retinae of three-month-old VPP animals. Intriguingly, in our transcriptome-wide analyses, we found more than 9000 dysregulated genes in retinae of VPP mice. The predominant changes in gene expression point towards induction of apoptosis, scar formation, neuroinflammation, and the upregulation of neuroprotective pathways like TGF-β, G-protein activated (e.g., endothelin), and VEGF signaling in VPP retinae. Using in situ hybridization combined with cell-type specific markers, we could show that regulatory factors such as *Ccl2*, *Edn2, Tgfbr2, Ednrb*, and *Vegfr2* were also expressed in glial cells in addition to neurons. Albeit the relevance of the identified pathways needs further investigation using, e.g., different (cell-type specific) knockout mouse lines, it is tempting to speculate that modulation of neuroinflammation and neuroprotective pathways in general or, e.g., in glial cells, is a promising target for the development of new therapeutic options to delay the degeneration of photoreceptors in diseases like RP.

## Figures and Tables

**Table 1:** **Enrichment analysis for dysregulated genes derived from the VPP RNAseq analysis.** Enriched pathways and potential upstream regulators were predicted using the indicated databases. For gene ontology enrichment, only the top five non-redundant significantly enriched biological process terms are shown. The numbers following the terms are the combined score as calculated by Enrichr. Only terms with a combined score >5 were considered.

Dysregulation Analysis	Enriched Pathways 1: BioPlanet 2019, 2: Reactome 2016, 3: NCI-Nature 2016	Gene Ontology Enrichment (Biological Process 2018)	Potential Regulators 1: ChEA 2016, 2: ENCODE TF ChIP-seq 2015
4620 upregulated genes (*p_ad_*_j_ < 0.05)	1: Platelet activation, signaling, and aggregation 98.77; Axon guidance 92.59; TGF-beta regulation of extracellular matrix 89.40; Integrin cell surface interactions 80.66; PI3K class IB pathway in neutrophils 69.39 2: Platelet activation, signaling, and aggregation 98.99; Integrin cell surface interactions 84.30; Hemostasis 73.18, EPH-Ephrin 62.44; Axon guidance 56.40 3: Integrin family cell surface interactions 61.52, S1P3 pathway 54.91, CXCR4-mediated signaling events 53.30, LPA receptor mediated events 52.65, S1P1 pathway 48.70	extracellular matrix organization 93.21, neutrophil activation involved in immune response 78.28, cellular response to cytokine stimulus 72.40, regulation of cell migration 69.84, vascular endothelial growth factor receptor signaling pathway 68.22	1: SUZ12 268.86, MTF2 160.06, WT1 96.47 2: EZH2 98.33, EP300 96.32, MYOD1 30.74
4636 downregulated genes (*p_adj_* < 0.05)	1: Messenger RNA processing 141.05, Visual signal transduction: rods 57.92, Global genomic nucleotide excision repair 45.63, Mitotic G2-G2/M phases 42.06, RNA polymerase II transcription 40.22, 2: Assembly of the primary cilium 107.65, mRNA splicing—major pathway 73.55, Activation of the phototransduction cascade 69.44, DNA repair 61.73, Cell cycle 58.51 3: Visual signal transduction: Rods 57.92, Fanconi anemia pathway 27.00, ATR signaling pathway 16.33, Regulation of Telomerase 13.58, ATM pathway 12.73, p38 MAPK signaling pathway 11.53	mRNA processing 168.47, DNA repair 93.72, cilium assembly 68.84, termination of RNA polymerase II transcription 55.78, rhodopsin mediated signaling pathway 48.90	1: CREM 212.80, FOXO3 172.49, KDM5B 152.04 2: KAT2A 233.47, GABPA 199.23, E2F4 196.15

**Table 2:** **Enrichment analysis for WGCNA modules derived from the VPP RNAseq analysis.** Enriched pathways and potential upstream regulators were predicted using the indicated databases. For gene ontology enrichment, only the top five non-redundant significantly enriched biological process terms are shown. The numbers following the terms are the combined score as calculated by Enrichr. Only terms with a combined score >5 were considered. Terms in bold font were also identified in the enrichment analysis of the dysregulated gene lists (combined score > 5). n.s. = no significant enrichment.

WGCNA Module	Enriched Pathways 1: BioPlanet 2019, 2: Reactome 2016, 3: NCI-Nature 2016	Gene Ontology Enrichment (Biological Process 2018)	Potential Regulators 1: ChEA 2016, 2: ENCODE TF ChIP-seq 2015
Pos1 (7705 genes)	**1:** Axon guidance 36.18; T helper cell surface molecules 32.25; Platelet activation, signaling, and aggregation 29.28; Alpha-V beta-3 integrin/OPN pathway 27.52; PI3K class IB pathway in neutrophils 25.55 **2:** Integrin cell surface interactions 31.84; Platelet activation, signaling, and aggregation 29.71; Ephrin signaling 27.88; Extracellular matrix organization 27.76; Signal amplification 24.50; Semaphorin interactions 20.52 **3:** Osteopontin-mediated events 27.52, Beta3 integrin cell surface interactions 25.55, S1P2 pathway 25.22, S1P3 pathway 24.00, LPA receptor mediated events 20.74	extracellular matrix organization 44.07, sprouting angiogenesis 27.06, ephrin receptor signaling pathway 24.92, response to cytokine 24.09, vascular endothelial growth factor receptor signaling 23.91	1: SUZ12 67.13, MTF2 35.24, JARID2 25.08 2: EZH2 14.15, EP300 6.68
Pos2 (560 genes)	**1:** Respiratory electron transport 148.50, Ketone body metabolism 70.09, Cap-dependent translation initiation 50.22, Nef-mediated CD8 downregulation 42.88, Cytoplasmic ribosomal proteins 39.30 **2:** Respiratory electron transport 128.38, Eukaryotic translation elongation 57.24, Nef mediated CD8 down-regulation 42.88, Orexin and neuropeptides FF and QRFP bind to their respective receptors 35.11, Ketone body metabolism 35.11 **3:** Validated nuclear estrogen receptor alpha network 15.80, JNK signaling in the CD4+ TCR pathway 14.61, PDGF receptor signaling network 11.03, Alpha-synuclein signaling 9.45, Visual signal transduction: Cones 6.22	respiratory electron transport chain 114.12, SRP-dependent cotranslational protein targeting to membrane 57.24, negative regulation of peptide 56.44, negative regulation of membrane potential 53.81, negative regulation of necroptotic process 53.81	1: EKLF 13.21, THRA 7.02, GATA1 6.80 2: HCFC1 6.41
Pos3 (272 genes)	**1:** Adrenoceptors 82.98, Phospholipase C delta-1 interactions in phospholipid-associated cell signalling 39.93, Serotonin and melatonin biosynthesis 39.93, FGFR1b ligand binding and activation 39.93, Pyrimidine biosynthesis 31.12 **2:** Adrenoceptors 82.98, Free fatty acid receptors 39.93, Arachidonate production from DAG 39.93, Serotonin and melatonin biosynthesis 39.93, FGFR1b ligand binding and activation 31.12 **3:** Signaling events mediated by the Hedgehog family 22.30, IL4-mediated signaling events 11.14, IL23-mediated signaling events 9.57, Circadian rhythm pathway 8.25, BMP receptor signaling 7.68	spinal cord dorsal/ventral patterning 365.39, osteoblast development 97.81, positive regulation of catenin import into nucleus 97.81, DNA replication-dependent nucleosome organization 71.54, septin ring assembly 39.82	1: FOXP1 17.13, BP1 6.21 2: n.s.
Neg1 (7248 genes)	**1:** Messenger RNA processing 59.45, Global genomic nucleotide excision repair 25.86, RNA polymerase II C-terminal domain phosphorylation and interaction with capping enzyme 22.43, Visual signal transduction: rods 21.50, Non-coding RNA metabolism 19.92 **2:** Assembly of the primary cilium 57.92, Processing of intronless pre-mRNAs 42.06, Processing of capped intron-containing Pre-mRNA 37.93, Homologous DNA Pairing and Strand Exchange 33.07, Activation of the phototransduction cascade 30.84 **3:** Fanconi anemia pathway 21.85, Visual signal transduction: Rods 21.50, ATR signaling pathway 5.94, Regulation of Telomerase 5.36, p38 MAPK signaling pathway 5.30	mRNA processing 70.46, DNA repair 38.32, ciliary basal body-plasma membrane docking 29.59, DNA-templated transcription, termination 26.90, histone lysine demethylation 22.88	1: FOXO3 51.83, YY1 27.21, CREB1 23.28 2: KAT2A 105.06, GABPA 89.64, E2F4 56.05
Neg2 (506 genes)	**1:** Signaling by FGFR1 fusion mutants 56.25, Activation of NOXA and translocation to mitochondria 16.74, Polo-like kinase 3 (PLK3) pathway 16.74, Cyclin B2-mediated events 16.74, Tachykinin receptors bind tachykinins 16.74 **2:** Signaling by cytosolic FGFR1 fusion mutants 61.27, Heme biosynthesis 25.15, Golgi cisternae pericentriolar stack reorganization 17.20, Defective ABCA3 causes pulmonary surfactant metabolism dysfunction type 3 (SMDP3) 16.74, Hyaluronan biosynthesis and export 16.74 **3:** PLK3 signaling events 16.74, Canonical NF-kappaB pathway 7.46, TNF receptor signaling pathway 5.68, TRAIL signaling pathway 5.22, Signaling mediated by p38-gamma and p38-delta 5.04	left/right pattern formation 49.64, viral RNA genome replication 21.96, DNA replication-independent nucleosome organization 19.36, mRNA splice site selection 18.87, positive regulation of vascular smooth muscle cell proliferation 17.20	1: n.s. 2: n.s.
Neg3 (586 genes)	**1:** Cytoplasmic ribosomal proteins 195.82; Spliceosomal assembly 176.30; Translation 169.55; Respiratory electron transport, ATP biosynthesis by chemiosmotic coupling, and heat production by uncoupling proteins 137.50; Apoptotic factor-mediated response 104.27 **2:** Eukaryotic translation initiation 251.77; Mitochondrial translation 208.25; Cytochrome c-mediated apoptotic response 170.73; rRNA processing 135.64; Respiratory electron transport, ATP synthesis by chemiosmotic coupling, and heat production by uncoupling proteins 129.02 **3:** PLK3 signaling events 13.51, DNA-PK pathway in nonhomologous end joining 10.86, HIV-1 Nef: Negative effector of Fas and TNF-alpha 7.31, C-MYC pathway 5.73	mitochondrial translation 175.43, mitochondrial electron transport, ubiquinol to cytochrome c 125.37, translation 123.40, rRNA metabolic process 122.84, activation of cysteine-type endopeptidase activity involved in apoptotic process by cytochrome c 104.27	1: JARID1A 89.00, ETS1 71.54, EKLF 44.12 2: EP300 54.49, GABPA 48.40, KAT2A 46.47

## Data Availability

The raw data files of the RNAseq data (Supplementary Table S2 and S3) are available from the authors upon request.

## Supplementary Materials

The following are available online at https://www.mdpi.com/article/10.3390/ijms22126307/s1, Figure S1: Apoptosis, retinal morphology/morphometry and the expression of neuroprotective factors in VPP and control mice, Figure S2: WGCNA analysis of VPP mice, Figure S3: Heatmaps of pathway analysis of transcriptomic changes in the VPP mice, Figure S4: Pathway analysis of transcriptomic changes in the VPP mice, Figure S5: The glial response to photoreceptor degeneration in the VPP model, Figure S6: z-stacks and corresponding 3D reconstructions, Table S1: Oligonucleotides for qPCR, Table S2: DEseq2 analysis, Table S3: WGCNA modules.

## Abbreviations

Bik	Bcl2-interacting killer
BMP	Bone morphogenetic protein
C1q	Complement component 1q
C3	Complement component 3
Casp1	Caspase 1
Ccl2	Chemokine (C-C motif) ligand 2
Ccl5	Chemokine (C-C motif) ligand 5
Cd3g	T-cell receptor T3 gamma chain
Cfh	Complement factor H
Cfi	Complement component factor i
Cr3	Complement receptor 3
Cxcl13	C-X-C motif chemokine ligand 13
Edn1	Endothelin 1
Edn2	Endothelin 2
Ednra	Endothelin receptor type a
Ednrb	Endothelin receptor type b
Fgf2	Fibroblast growth factor 2
Fgl2	Fibrinogen-like 2
GCL	Ganglion cell layer
Gfap	Glial fibrillary acidic protein
Glb1l3	Galactosidase beta 1 like 3
Gnb2l1	Guanine nucleotide binding protein subunit beta2 like 1
GS	Glutamine synthetase
Hdc	Histidine decarboxylase
Iba1	Ionized calcium-binding adapter molecule 1
Il-1	Interleukin-1
INL	Inner nuclear layer
IPL	Inner plexiform layer
Kdr	Kinase insert domain receptor
Lif	Leukemia inhibitory factor
Lcn2	Lipocalin 2
ONH	Optical nerve head
ONL	Outer nuclear layer
OPL	Outer plexiform layer
Prss56	Serine protease 56
RNAseq	Next generation RNA sequencing
RP	Retinitis pigmentosa
RPE	Retinal pigment epithelium
Serpina3n	Serine protease inhibitor A3N
TGF	Transforming growth factor
Tgfbr1	Transforming growth factor -ß receptor type 1
Tgfbr2	Transforming growth factor -ß receptor type 2
Ubc	Ubiquitin C
VEGF	Vascular endothelial growth factor
Vegfr1	Vascular endothelial growth factor receptor type 1
Vegfr2	Vascular endothelial growth factor receptor type 2
WGCNA	Weighted correlation network analysis
