# Supplementary material for: Transcriptional Profiling Identifies Upregulation of Neuroprotective Pathways in Retinitis Pigmentosa
Source: Int J Mol Sci. 2021 Jun 11;22(12):6307. doi: 10.3390/ijms22126307 (PMC8231189; doi:10.3390/ijms22126307)
Supplement: Supplementary file 1 [file ijms-22-06307-s001.zip › Table1_suppl_Primer.pdf]

Gene	Species	Orientation	Sequence 5´- 3´
Ccl2	*Mus musculus*	sense	catccacgtgttggctca
		antisense	gatcatcttgctggtgaatgagt
Edn2	*Mus musculus*	sense	acctcctccgaaagctgag
		antisense	tttcttgtcacctctggctgta
Fgf2	*Mus musculus*	sense	ccaaccggtaccttgctatg
		antisense	ccagtcgttcaaagaagaaacac
Gfap	*Mus musculus*	sense	tcgagatcgccacctacag
		antisense	gtctgtacaggaatggtgatgc
Gnb2l1	*Mus musculus*	sense	tctgcaagtacacggtccag
		antisense	acgatgatagggttgctgc
Iba1	*Mus musculus*	sense	ggatttgcagggaggaaaag
		antisense	tgggatcatcgaggaattg
Lif	*Mus musculus*	sense	aaacggcctgcatctaagg
		antisense	agcagcagtaagggcacaat
Ubc	*Mus musculus*	sense	gtctgctgtgtgaggactgc
		antisense	cctccagggtgatggtctta

Table 1: Oligonucleotides for qPCR
