# Supplementary figures and images for: Transcriptional Profiling Identifies Upregulation of Neuroprotective Pathways in Retinitis Pigmentosa
Source: Int J Mol Sci. 2021 Jun 11;22(12):6307. doi: 10.3390/ijms22126307 (PMC8231189; doi:10.3390/ijms22126307)

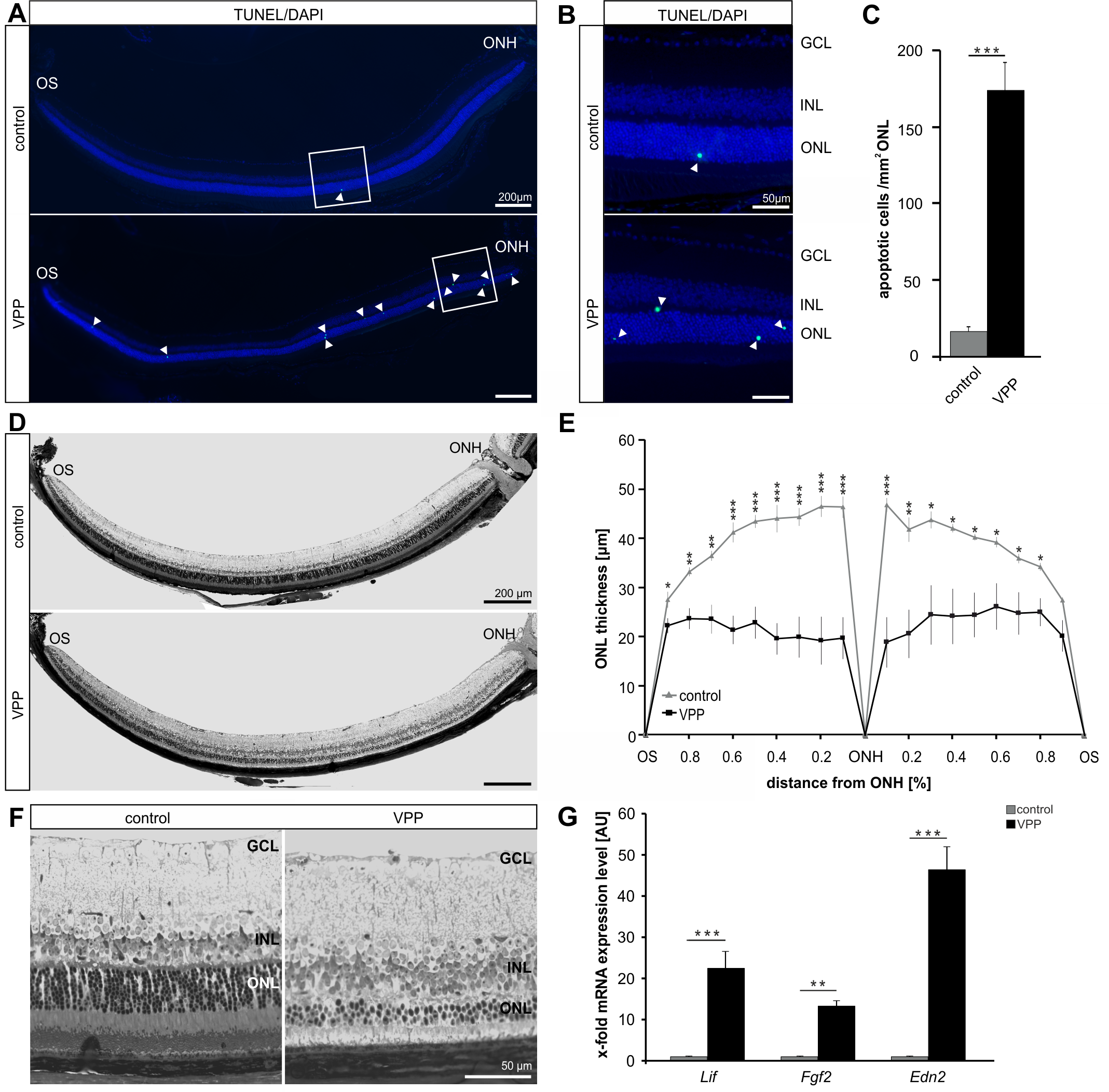

Supplement: Supplementary file 1 [file ijms-22-06307-s001.zip › Fig_suppl_1.tif]

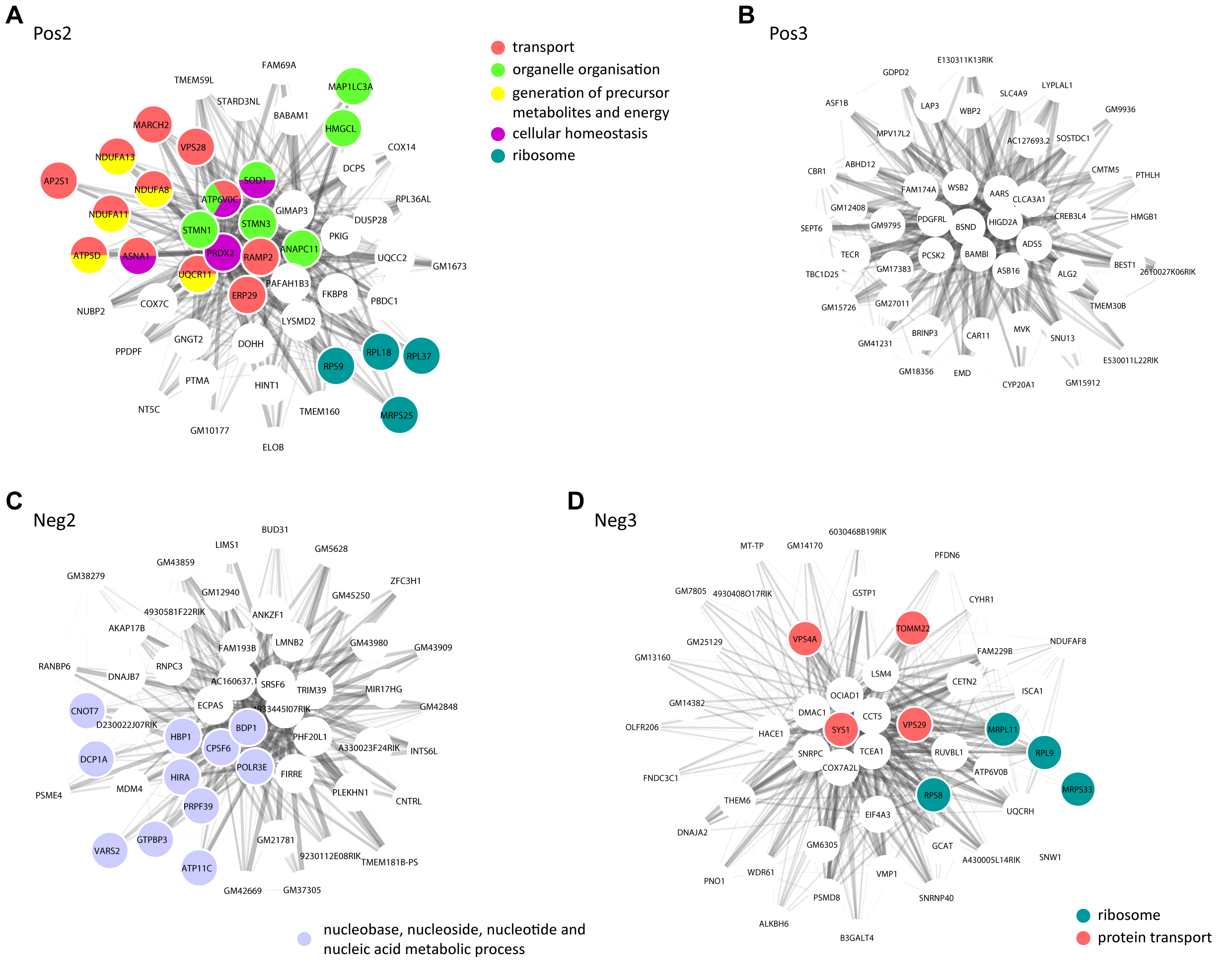

Supplement: Supplementary file 1 [file ijms-22-06307-s001.zip › Fig_suppl_2_networks-01.tif]

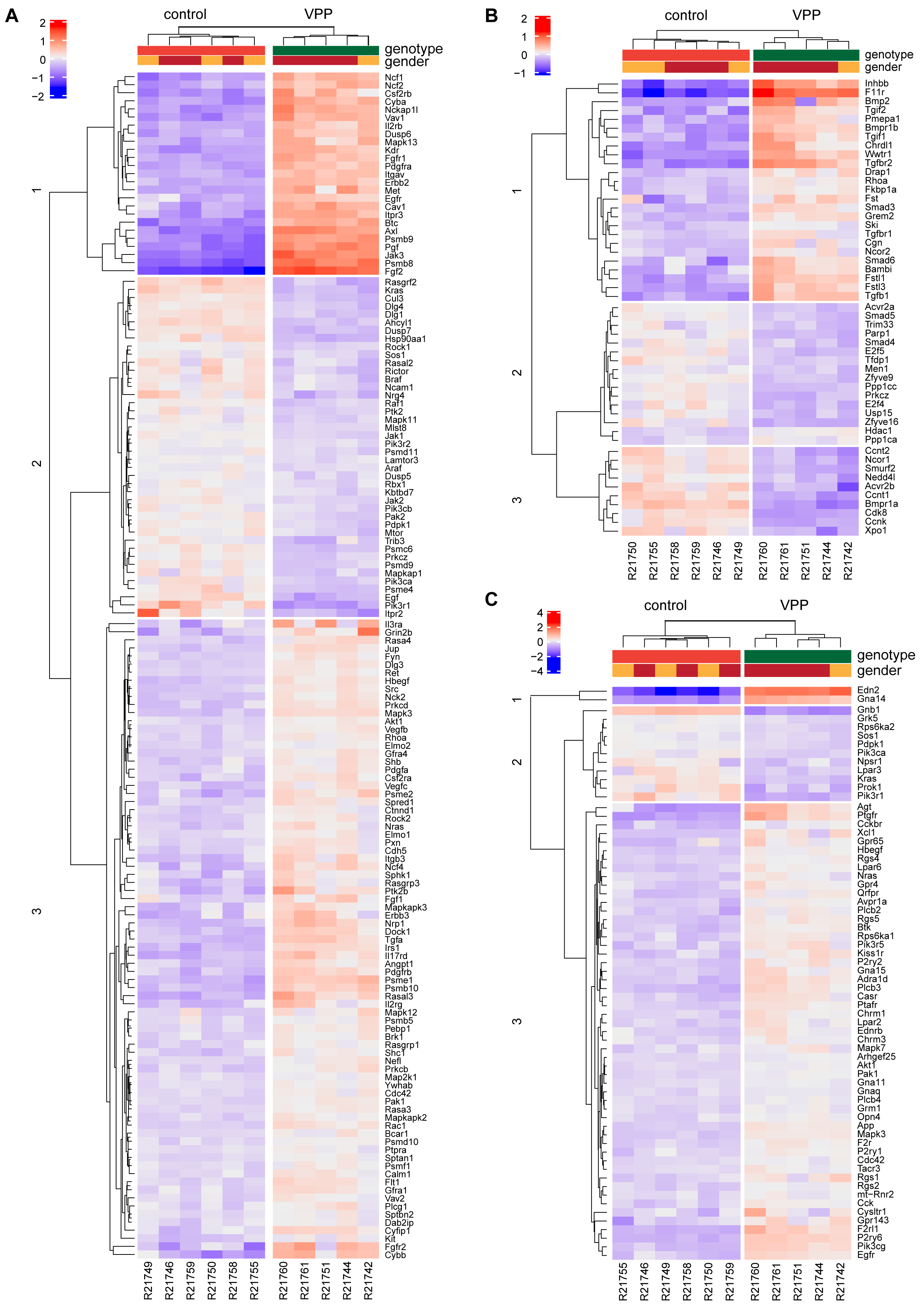

Supplement: Supplementary file 1 [file ijms-22-06307-s001.zip › Fig_suppl_3_heatmaps-01.tif]

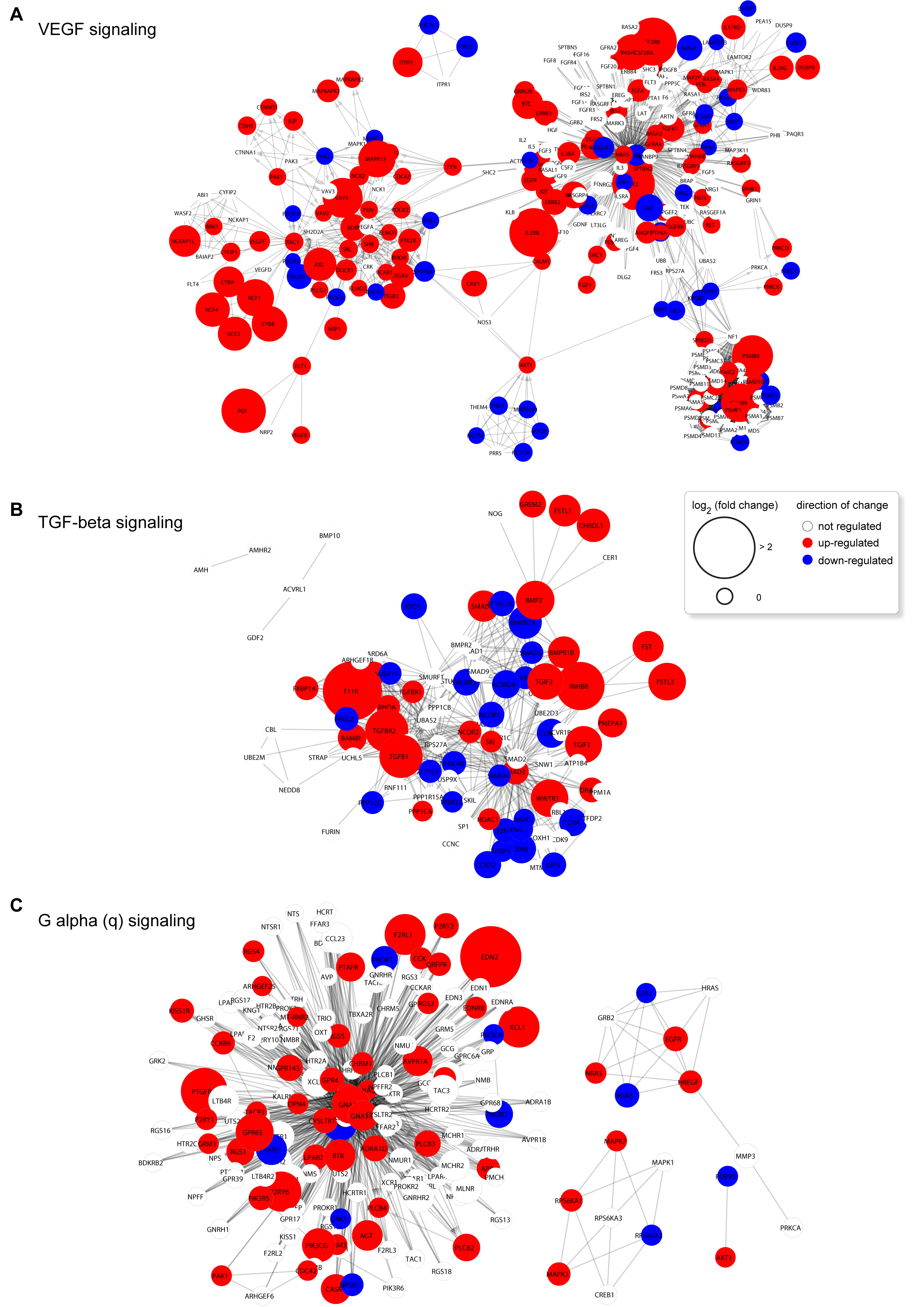

Supplement: Supplementary file 1 [file ijms-22-06307-s001.zip › Fig_suppl_4_pathways-01.tif]

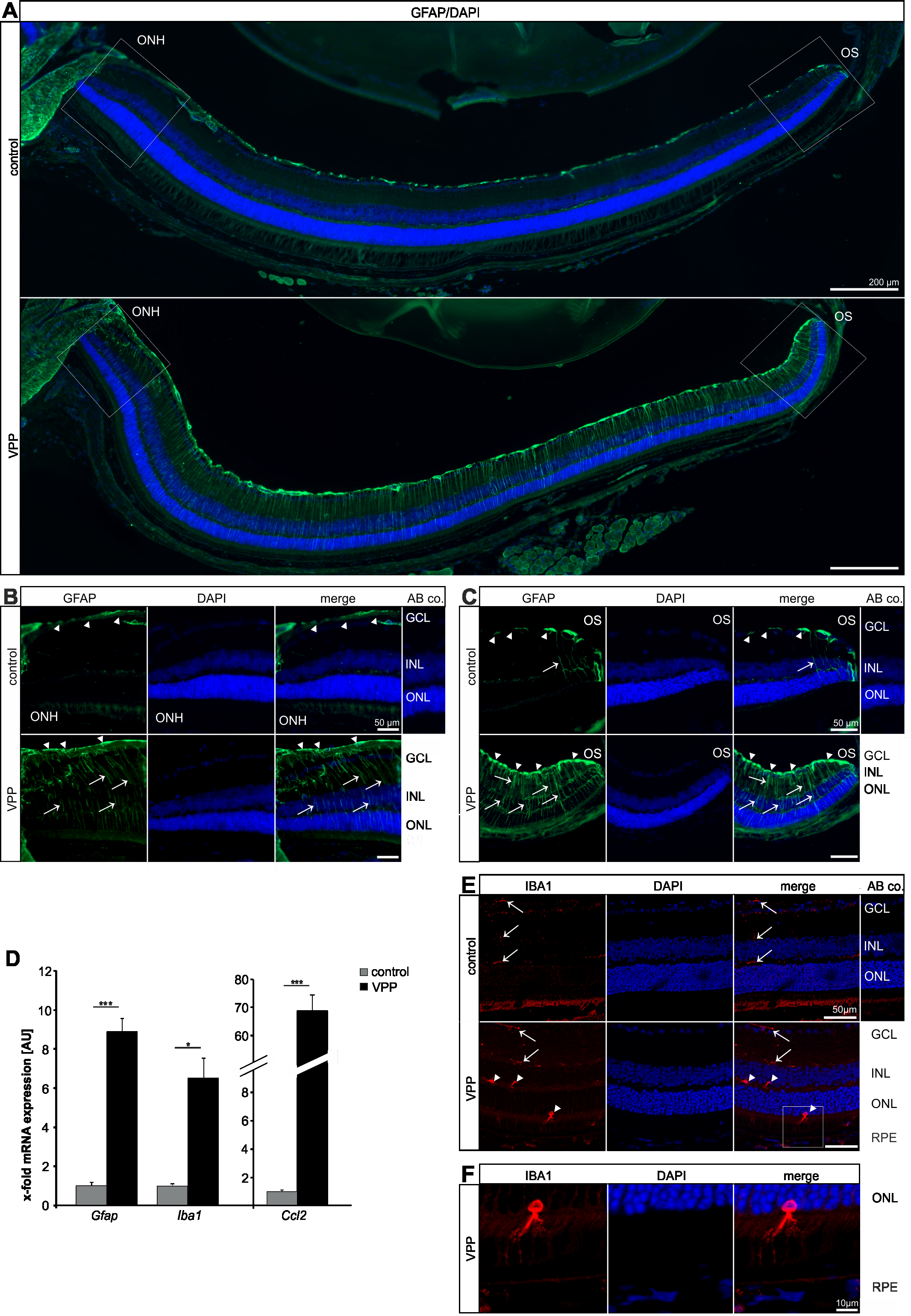

Supplement: Supplementary file 1 [file ijms-22-06307-s001.zip › Fig_suppl_5_GFAP_neu.tif]

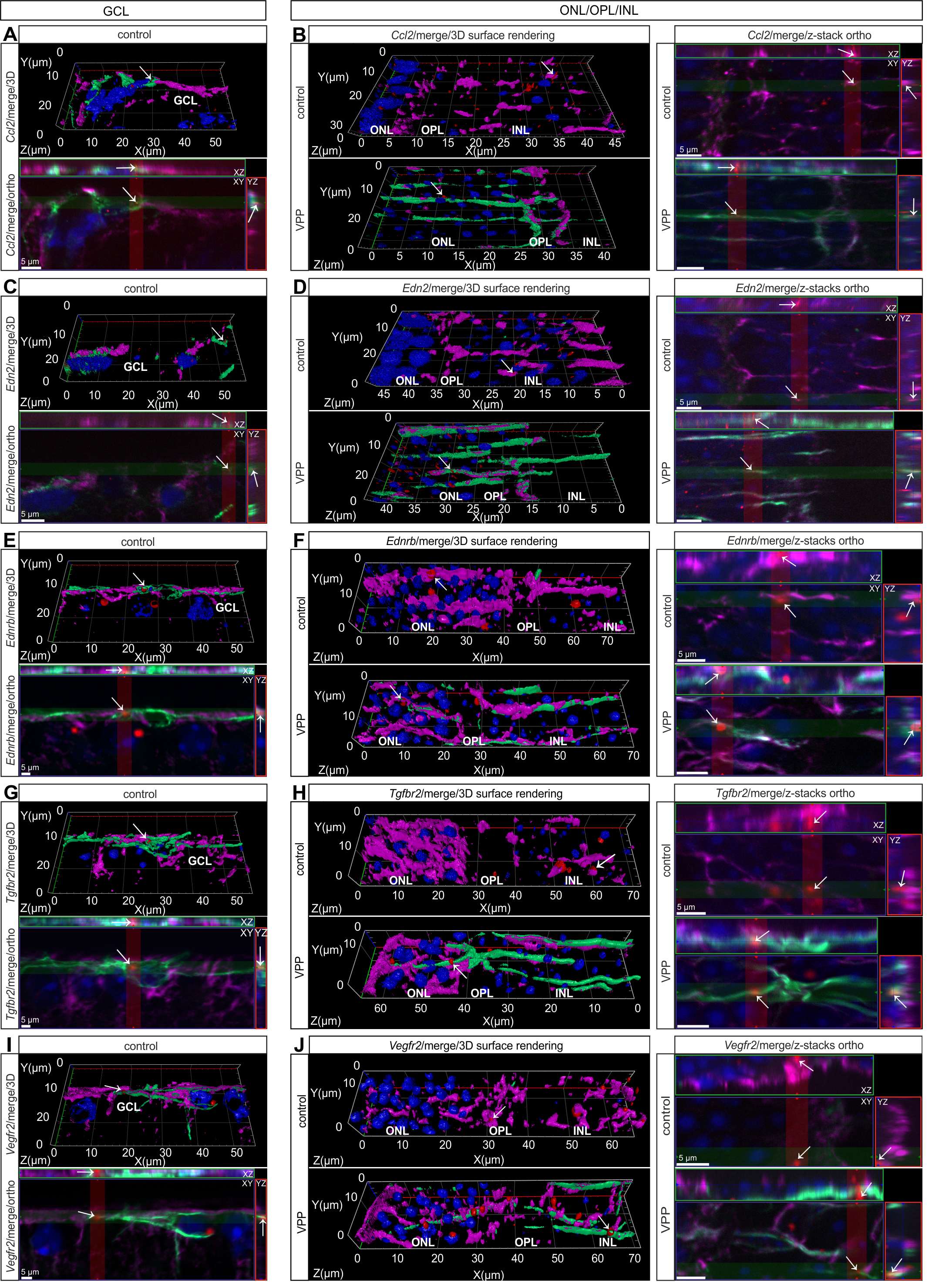

Supplement: Supplementary file 1 [file ijms-22-06307-s001.zip › Fig_suppl_6_ortho.tif]
